# Chimeric oncolytic Ad5/3 virus replicates and lyses ovarian cancer cells through desmoglein‐2 cell entry receptor

**DOI:** 10.1002/jmv.25677

**Published:** 2020-02-03

**Authors:** Lukasz Kuryk, Anne‐Sophie W. Møller

**Affiliations:** ^1^ Clinical Science Targovax Oy Helsinki Finland; ^2^ Department of Virology National Institute of Public Health – National Institute of Hygiene Warsaw Poland; ^3^ Clinical Science Targovax ASA Oslo Norway

**Keywords:** CAR, CD46, desmoglein‐2, oncolytic adenovirus, ovarian cancer

## Abstract

Despite new therapies, the estimated 229 875 women living with ovarian cancer have a 5‐year survival rate of 47.6%. This cavity‐localized cancer lends itself to local administration of modalities, such as the oncolytic adenovirus (Ad) Ad5/3‐D24‐granulocyte‐macrophage colony‐stimulating factor virus (ONCOS‐102). Its repeated administration to a patient with chemotherapy‐refractory ovarian cancer induced CD8+ antitumor immune responses with the overall survival reaching 40 months. Here we probe the dominant receptor used by ONCOS‐102 in four established epithelial ovarian cancer cell lines. Ad3 can use the desmoglein‐2 (DSG2) and CD46 receptors on susceptible cells. DSG2 was nearly absent in A2780 cells but was expressed in more than 90% of OAW42, OVCAR3, and OV‐90 cells. After 96 hours, ONCOS‐102 treatment showed significant oncolytic activity (≧50%) in OAW42, OVCAR3, and OV‐90 cells, but minimal activity in A2780 cells, suggesting DSG2 as the dominant receptor for ONCOS‐102. Furthermore, retrospective analyses of phase I clinical trial of ONCOS‐102 treatment of 12 patients with varied tumors indicated a correlation between viral genomes in blood and DSG2 RNA expression. These data support the role of DSG2 expression on cancer cells in virus infectivity and the continued development of ONCOS‐102 for ovarian cancer treatment.

## Research Highlights

- These data support the role of DSG2 expression on cancer cells in virus infectivity and the continued development of ONCOS‐102 for ovarian cancer treatment.

## 1 INTRODUCTION

Ovarian cancer is diagnosed at an advanced stage in most patients (59%),^1^ and metastases most often occur on the omentum and other organs in the peritoneal cavity. Despite the development of new agents, the mean survival of ovarian cancer patients has remained between 41.2 to 47.8 months since 1987.^2^ An estimated 229 875 women in the United States had ovarian cancer in 2016, and their 5‐year survival rate in 2016 was 47.6%.^3^ This cavity‐localized cancer lends itself to local administration of modalities, such as oncolytic adenoviruses, that is, adenoviruses that replicate in and lyse tumor cells but not normal cells.^4^, ^5^, ^6^, ^7^, ^8^, ^9^, ^10^, ^11^, ^12^, ^13^, ^14^ For example, nine administrations of an oncolytic adenovirus, ONCOS‐102, into a patient with chemotherapy‐refractory ovarian cancer in a phase I trial induced and progressively enhanced CD8+ infiltration in the treated tumor lesions.^15^ Systemic CD8+ T cell responses against several tumor antigens were detected from 8 to 113 days after treatment initiation.^15^ Herein, we investigate the susceptibility of established ovarian cancer cell lines with varied adenovirus (Ad) cell entry receptor expression to the oncolytic activity of ONCOS‐102.

Because primary ovarian cancer cells have a variable expression of the adenovirus 5 (Ad5) coxsackie and adenovirus receptor (CAR), ranging from 30% to 99% of cells,^16^ many groups have pursued the development of alternate serotypes of adenoviral vectors such as Ad3, or they have engineered chimeric Ad5 fiber knob proteins to bind to an alternate receptor.^5^, ^17^, ^18^ CD46 is considered a receptor for Ad3, Ad7, Ad11, and Ad35^19^; it functions as a complement regulatory protein. Most epithelial ovarian cancer (EOC) samples from a primary laparotomy and secondary cytoreduction procedures stained positive for CD46 (60% and 70%, respectively).^20^ CD46 was highly expressed in 100% of primary EOC cancer lines (5 of 5).^21^ Desmoglein‐2 (DSG2) is also a receptor for Ad3, Ad7, and Ad11.^22^ DSG2 is overexpressed in many types of ovarian cancers.^23^ We and others have engineered Ad5 with chimeric Ad5/3 fiber knobs to target epithelial cancers^18^, ^24^ that overexpress the DSG2 and/or the CD46 receptors.

ONCOS‐102 has three modifications that can contribute to its safety and its efficacy against ovarian cancer.^18^ Its chimeric Ad5/3 fiber knob changes the binding specificity of the virus: instead of binding to the CAR, this chimeric Ad5/3 adenovirus targets the frequently overexpressed membrane proteins DSG2 and CD46. The replication of ONCOS‐102 is restricted to tumor cells with an altered Rb pathway by its 24 bp deletion in the E1A gene. Its expression of granulocyte‐macrophage colony‐stimulating factor (GM‐CSF) can augment the immunostimulatory milieu in the infected tumor.^5^, ^15^, ^25^, ^26^, ^27^

Here, we investigated the oncolytic activity of ONCOS‐102 in vitro in four ovarian adenocarcinoma cell lines that differ in expression of DSG2 but have a similar expression of CD46 or CAR to explore the prominent receptor for transduction. To assess the role of expression of the DSG2 and CD46 receptors on ONCOS‐102 treatment of patients with solid tumors from a previously reported phase I trial,^28^ we retrospectively investigated DSG2 and CD46 RNA expression levels in patient tumor samples and their relationship with viral load in blood, and with the number of tumor‐infiltrating leukocytes (TILs).

## 2 MATERIALS AND METHODS

### 2.1 Ovarian cancer cell lines

Two human ovarian carcinoma cell lines, A2780 (93112519‐1VL/lot:16L020; Sigma) and OAW42 (85073102‐1VL/lot:13F010; Sigma) were purchased from Sigma. Two human ovarian carcinoma cell lines OV‐90 (ATCC CRL‐117321/lot: 63990123) and OVCAR3 (ATCC HTB‐161) were purchased from ATCC. All cell lines were cultured in the indicated media with the indicated 10% to 20% fetal bovine serum (FBS), and 1% penicillin/streptomycin (Table S1). Cells were passed at 80% of the confluence with trypsin‐EDTA and the doubling times ranged from 1 day to 7 days: A2780, 1 day; OAW42, 2 days; OVCAR3, 3 days; OV‐90, 7 days.

### 2.2 Receptor expression

Flow cytometry was used to quantify the cell surface expression of the three receptors for adenovirus: CAR, CD46, and DSG2. DSG2 and CD46 cell surface expression were measured on the four human ovarian carcinoma cell lines (1 × 10^5^ cells/cell line) after a 30 minutes incubation at 4°C with PE‐conjugated anti‐DSG2 antibodies (12–9159‐42; Thermo Fisher Scientific) or PE‐Vio770‐conjugated anti‐CD46 antibodies (130‐104‐559; Miltenyi Biotec), respectively, and washed with PBS. CAR receptor cell surface expression were measured on the four human ovarian carcinoma cell lines (1 × 10^5^ cells/cell line) after a 30 minutes incubation at 4°C with the primary anti‐CAR rabbit polyclonal antibody (PA5–12476; Thermo Fisher Scientific), washed and labeled with anti‐rabbit Alexa‐Fluor 488 secondary antibody (Ab150077; Abcam) for 30 minutes at 4°C, and washed in PBS. Results were acquired on the Attune Nxt Flow cytometer on at least 10^4^ events in duplicate in two independent experiments.

### 2.3 ONCOS‐102 preparation and treatment

Adenovirus ONCOS‐102 is a class II genetically modified microorganism. The engineering of ONCOS‐102 has been described previously.^18^ ONCOS‐102 was produced and stored at −80°C, as previously described.^29^, ^30^ Briefly, the concentration of total viral particles (VP) was assessed by measurements with UV/Vis spectrophotometry at 260 and 280 nm. The VP was calculated with the formula: OD_260_ reading × dilution factor × 1.1 × 10^12^ particles = number of particles per mL of sample.

### 2.4 Cell viability

Oncolytic efficacy was determined with the MTS cell viability assay (ab197010; Abcam) at 72 and 96 hours postinfection. Briefly, the human ovarian cell lines were plated at 2 × 10^3^ cells/well in 96 flat bottomed tissue culture plates and incubated at 37°C. Cells were either incubated with zero viruses (control) or infected with ONCOS‐102 at 0.1, 1, 10, 100, or 1000 VP/cell in triplicate. Because the cultured cells were not confluent during infection, few tight junctions would have been present and thus both DSG2 and CD46 receptors would have been accessible during infection.

### 2.5 Retrospective analysis of samples from ONCOS‐102 treated patients (NCT01598129)

Retrospective analyses were performed with previously obtained data from patients who had enrolled and participated in the escalating dose phase I clinical trial, NCT01598129.^28^ Briefly, 12 patients with various types of solid tumors had been injected intratumorly and intravenously with ONCOS‐102 on days 1, 4, 8, 15, 29, 57, 85, 113, and 141.^28^ The low, medium, and high dose groups had received 3 × 10^10^, 1 × 10^11^, and 3 × 10^11^ VP/injection, respectively, at each time point.^28^ The tumor samples had been harvested at baseline, 1 month, and 2 months postinitiation of ONCOS‐102 treatment.^28^ Blood samples had been collected before each treatment, 6 and 24 hours after each treatment, then processed, and archived.^28^ DSG2 and CD46 RNA expression levels of the tumor samples had been obtained by microarray but had not been previously reported.^28^ The quantity of ONCOS‐102 viral genomes in the blood samples had been measured by real‐time polymerase chain reaction and previously reported. The different leukocyte populations in the tumor biopsies, called TILs, had been determined by immunohistochemistry performed on formalin‐fixed and paraffin‐embedded tissues as previously described and reported.^28^

### 2.6 Data analysis

All variables were analyzed by using GraphPad Prism (v8) software. The correlation was calculated using the nonparametric Spearman test (two‐tailed, 95% confidence interval). Statistical significance for in vitro studies was performed by two‐way analysis of variance.

## 3 RESULTS

### 3.1 Receptor expression

**Figure 1:**
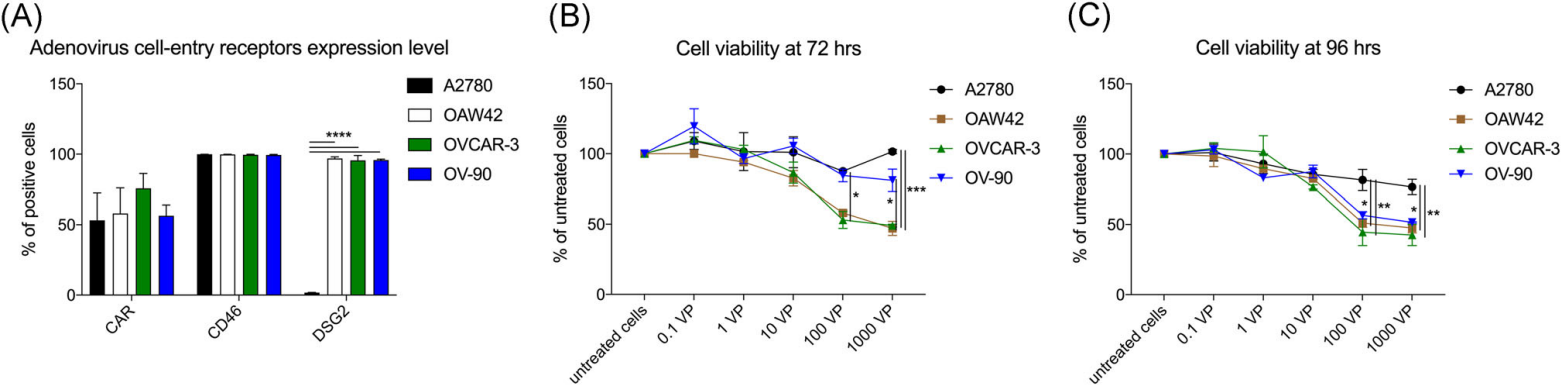
EOC receptor expression and sensitivity to oncolytic activity to ONCOS‐102 treatment. A, Flow cytometry analyses of CAR, CD46, and DSG2 receptor expression on ovarian cancer cells. At least 10^4^ events were analyzed for each marker and cell line. Results represent the mean ± SEM of at least two independent experiments. Cell viability at (B) 72 hours or (C) 96 hours after ONCOS‐102 treatment in five different concentrations was assessed with the MTS assay. Results are expressed as the mean percent of untreated cells ± SEM. Data represents a pool of two independent experiments run in triplicate. CAR, coxsackie and adenovirus receptor; DSG2, desmoglein‐2; EOC, epithelial ovarian cancer

Both A2780 and OAW42 ovarian carcinoma cell lines are categorized as nonserous adenocarcinoma cell lines and OVCAR3 as high‐grade serous by genomic profiles.^31^ OV‐90 is classified as undifferentiated adenocarcinoma. These four ovarian carcinoma cell lines highly expressed CD46, a major receptor for the Ad3 serotype (Figure 1A). DSG2 was expressed in 1.8% of A2780 cells, whereas other cell lines had a significantly higher expression of 97%, 96%, and 96% for OAW42, OVCAR3, and OV‐90, respectively. Presence of CAR adenovirus receptor on the cell surface was observed in 53% ± 19% of A2780 cells, 58% ± 18% of OAW42, 76% ± 11% of OVCAR3, and 56% ± 8% of OV‐90 cells. The mean fluorescence intensity for CD46 was 99.88 ± 0.23 on A2780 cells, 99.85 ± 0.05 on OAW42 cells, 99.5 ± 0.4 on OVCAR3 cells, and 99.4 ± 0.4 on OV‐90 cells.

### 3.2 Oncolytic activity

The efficacy of ONCOS‐102 treatment was determined at 72 and 96 hours postinfection with the MTS cell viability assay. At 72 hours post‐ONCO‐102 treatment (Figure 1b and Table 1), ONCOS‐102 treatment (1000 and 100 VP/cell), the cell viability of OAW42 (53%) and OVCAR3 (51%) was significantly reduced as compared with their respective untreated cells (*P* < .01). ONCOS‐102 also modestly but not significantly reduced cell viability in OV‐90 (19%) (Figure 1B and Table 1). In contrast, ONCOS‐102 treatment did not reduce the viability of the A2780 cells at 72 hours (Figure 1B and Table 1).

**Table 1:** Oncolytic activity of ONCOS‐102

Cell lines	ONCOS‐102, VP/cell	Mean difference from untreated cells (%)	95% Confidence interval of difference (lower, upper)	*P* value
72 h				
A2780	100	12.5	−20.77, 45.77	.9938
	1000	−1.5	−34.77, 31.77	.9999
OAW42	100	42	17.73, 84.27	.0045
	1000	53	19.73, 86.27	.0002
OVCAR3	100	47.0	13.73, 80.27	.0011
	1000	51.0	17.73, 84.27	.0003
OV‐90	100	15.5	−14.27, 52.27	.9453
	1000	19.0	−14.27, 52.27	.7693
96 h				
A2780	100	18.5	−10.33, 47.33	.5947
	1000	23.5	−5.331, 52.33	.2175
OAW42	100	49.0	20.17, 77.83	<.0001
	1000	52.5	23.67, 81.33	<.0001
OVCAR3	100	55.5	23.67, 81.33	<.0001
	1000	57.5	28.67, 86.33	<.0001
OV‐90	100	43.5	14.67, 72.33	.0004
	1000	48.5	19.67, 77.33	<.0001

ONCOS‐102 treatment (1000 VP/cell) at 96 hours (Figure 1C, Table 1) significantly reduced the cell viability of OAW42 (52%), OVCAR3 (57%), and OV‐90 (49%) compared with their respective untreated cultured cells (*P* > .0001; Figure 1C). Similar results were obtained with ONCOS‐102 treatment of 100 VP/cell in these cell lines (*P* < .0004; Figure 1C). In contrast, ONCOS‐102 treatment (1000 VP/cell) had modestly but not significantly reduced the viability of the A2780 cells by 23% at 96 hours as compared with the untreated A2780 cells (*P* > .20). These data support that the ONCOS‐102 infection of ovarian epithelial carcinoma cells primarily relied on at least initial binding to DSG2 for its uptake.

### 3.3 Association of DSG2 RNA expression with higher ONCOS‐102 genome copies in a clinical trial

**Figure 2:**
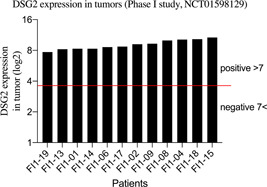
RNA expression levels of DSG2 in tumor samples from 12 patients with tumors of various origins who received repeated administrations of ONCOS‐102 administration in a previously described phase I clinical trial (NCT01598129).^28^ RNA expression had previously been determined via microarray but was not reported.^28^ Two patients (Fl1‐01 and Fl1‐19) had chemotherapy‐refractory ovarian cancer. DSG2, desmoglein‐2

To investigate whether DSG2 expression level may affect ONCOS‐102 replication in patients with tumors, we retrospectively analyzed data from tumor biopsy and blood samples from the previously reported ONCOS‐102 phase I clinical trial NCT01598129.^28^ The 12 enrolled patients had a solid tumor of varying origin that was refractory to standard treatments.^28^ Patients Fl1‐19 and Fl1‐01 had ovarian cancer.^28^ The DSG2 expression level measured by microarray was positive for the 12 patients (Figure 2).

**Figure 3:**
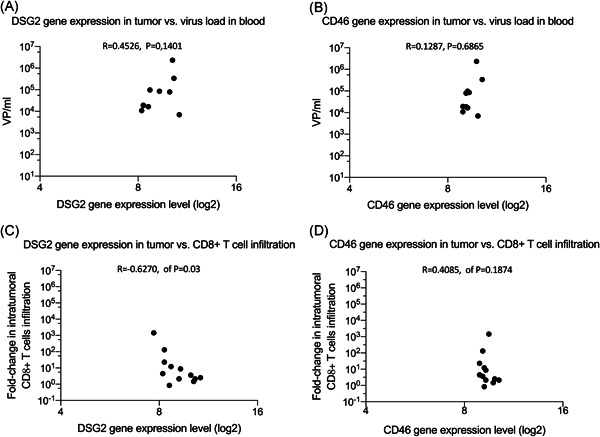
Relationships of DSG2 and CD46 RNA expression levels with the viral titer in blood and with the peak fold change in tumor‐infiltrating leukocytes (TILs). Blood and tumor samples were obtained from the 12 patients with tumors of various origins who had been repeatedly administered ONCOS‐102 in the previously described phase I clinical trial (NCT01598129).^28^ Two patients (Fl1‐01 and Fl1‐19) had chemotherapy‐refractory ovarian cancer. Comparison of the retrospective analysis of (A) DSG2 and (B) CD46 RNA expression levels previously performed by microarray on the baseline tumor samples with the number of ONCOS‐102 genomes found in the blood before the second ONCOS‐102 injection (day 4). Comparison of the retrospective analysis of (C) DSG2 and (D) CD46 RNA expression levels with the peak change in TILs that was detected by immunohistochemistry in either the 1‐ or 2‐month tumor samples from patients enrolled in phase I clinical trial.^28^ DSG2, desmoglein‐2

The DSG2 RNA expression levels in the tumor tissue from the 12 patients positively correlated, but not significantly with the number of viral genomes in the patients' blood on day 4 (Spearman's rank correlation, *R* = .4526; *P* = .1401; Figure 3A). CD46 RNA expression levels in the tumor tissues from the 12 patients had a weak correlation with the number of viral genomes in the patients' blood on day 4 (Spearman's rank correlation, *R* = .1287; *P* = .6865; Figure 3B).

Interestingly, DSG2 RNA expression levels in the tumor tissue from the 12 patients were significantly negatively correlated with the fold change in the peak number of CD8+ TILs at 1 or 2 months (Spearman's rank correlation, *R* = −.6270; *P* = .03; Figure 3C). DSG2 expression levels appeared to be negatively associated with fold change in intratumoral CD8+ cells. A positive correlation (not significant) was observed between CD46 RNA expression levels in the tumor tissue from the 12 patients and the number of CD8+ TILs (Spearman's rank correlation, *R* = .4085; *P* = .1874; Figure 3D).

As an update to the survival data from the phase I trial,^28^ the ovarian cancer patient, Fl1‐19, has survived 40 months. The peak fold change of TILs at 1 month or 2 months, as originally reported^28^ remained significantly associated with the updated lengths of overall survival (Spearman's rank correlation, *R* = .7180; *P* = .010; data not shown).

## 4 DISCUSSION

Ovarian cancer treatment, despite new modalities, has only modestly increased the 5‐year survival rate in the last decade.^3^ Thus, novel approaches including the development of immunogenic oncolytic adenoviruses, are warranted.^32^, ^33^ Efficient uptake and replication in the EOC cells are essential for oncolytic activity and necessitates the identification of the dominating receptor used for adenoviral uptake. Here we show that low/absence DSG2 expression significantly reduced the oncolytic activity of ONCOS‐102. ONCOS‐102 showed significant oncolytic activity against three of the four EOC cell lines (OAW42, OVCAR3, and OV‐90 but not A2780). Ad3 can utilize CD46^19^ and DSG2 receptors for viral binding and entry to its target cells.^22^ Ad5 vectors with the chimeric Ad5/3 fiber knob modification, including ONCOS‐102, are thought to use both CD46 and DSG2 to bind to and infect cancer cells. Since more than 95% of cells of the four cell lines expressed CD46, the oncolytic activity of ONCOS‐102 did not correlate with the CD46 expression levels. Instead, the high oncolytic activity of ONCOS‐102 correlated with the high DSG2 expression levels in the three EOC cell lines. Previously it has been shown that Ad3 infection of A549 lung cancer cells and human 16HBE14o bronchial cells were mainly mediated via binding with DSG2 and only about 10% occurred via the CD46 receptor,^34^ which supports our results. The primary ovarian tumor samples from the two patients in our phase I trial^28^ expressed DSG2 RNA, in agreement with DSG2 RNA expression in nearly all primary EOC samples.^23^, ^35^, ^36^ The DSG RNA expression was positively correlated (*R* = .4526) with the viral genomes released 3 days postinjection in ONCOS‐102‐treated patients with tumors of varied origin,^28^ although the correlation did not reach significance (*P* = .1401), possibly due to the cohort, was comprised mostly of other primary tumors (10 of 12). These data further support the role of DSG2 expression as a major receptor for ONCOS‐102 replication in primary tumor cells, including EOC cells.

Expression of adenoviral receptors varies in primary isolates of EOC and established cell lines. CAR cell surface proteins were detected on average in 55% to 75% of the cells of the four established EOC cell lines (A2780, OAW42, OVCAR3, and OV‐90), in agreement with its expression in 30% to 99% of cells from seven primary EOC, with a median of 92% positive CAR cells (interquartile range 40%, 98%).^16^ Because of the Ad5/3 fiber modification, ONCOS‐102 is not expected to bind to CAR. CD46 expression was expressed in 99% to 100% of seven primary EOC cells,^16^ similar to our findings of more than 90% CD46 positive cells in the four EOC cell lines used herein. CD46 is often located within tight junctions and less accessible than DSG2 in many cells, including ovarian cancer.^37^ In the ONCOS‐102‐treated patients with tumors of varied origin,^28^ the CD46 RNA expression showed only a weak positive correlation with the viral genomes released 3 days postinjection: the correlation was not significant (*R* = .1287; *P* = .6865). Whereas binding of Ad3 fiber knobs to DSG2 can open tight junctions and expose CD46 for interaction with Ad3,^38^ these data suggest that DSG2 plays a more predominant role than CD46 in ONCOS‐102 viral attachment and infection of EOC cells. Elucidation of the predominant receptor for Ad5/3 oncolytic viruses in primary tumors can support the prescreening of receptor expression in individual tumors, thereby advancing personalized cancer therapy. DSG2 overexpression is associated with tumor progression in hepatocellular carcinoma (HCC)^39^ and malignant melanoma (MM).^40^ DSG2 overexpression in HCC is an independent risk factor for reduced overall survival.^39^ In MM, DSG2 overexpression may promote vasculogenic mimicry via cell‐cell interactions and adhesion, but not viability, motility, and proliferation.^40^ Therefore, cancer cells expressing DSG2 seem to be a good target for treatment with chimeric oncolytic adenoviruses such as ONCOS‐102.

### 4.1 Limitations

First, although the oncolytic activity of ONCOS‐102 was not detected in A2780 cells, A2780 is susceptible to the oncolytic activity of replication‐selective Ad5 agents.^41^ We tested the oncolytic activity of ONCOS‐102 on four established EOC cell lines herein which was associated with DSG2 expression levels on the EOC cell lines. Second, because the phase I study had enrolled only two patients with EOC, we retrospectively compared the DSG2 and CD46 RNA expression levels with viral load in blood from 12 ONCOS‐102‐treated patients with various solid tumors. Although these 12 samples were positive for both DSG2 and CD46 expression, some primary EOC samples may not test positive for DSG2 and/or CD46, as previously reported.^20^ Third, repeated ONCOS‐102 administration induced significant increases in TILs in both patients with chemotherapy‐refractory ovarian cancer in a phase I trial^28^ which supports subsequent larger trials in ovarian cancer patients.

Furthermore, several primary ovarian cancer isolates were susceptible to a different conditional replicative Ad5/35 chimeric fiber knob vector^16^, ^42^ that did not express an exogenous immunogenic GM‐CSF.

Therefore, oncolytic adenoviruses with a modified knob can successfully infect cancer cells expressing DSG2 and subsequently induce the development of antitumor immune responses in 11 of 12 patients^28^ and clinical efficacy.

## 5 CONCLUSIONS

In conclusion, these data showed that the dominant receptor for ONCOS‐102 binding and replication was DSG2 in these ovarian cancer cell lines. Furthermore, these data support the continued development of ONCOS‐102 for the treatment of ovarian cancer. We speculate that patients with ovarian cancer expressing DSG2 may be more susceptible to the oncolytic activity of ONCOS‐102 and other oncolytic AD5/3 vectors than those with EOC without detectable DSG2 expression.

## CONFLICT OF INTERESTS

LK and A‐SWM are employees and/or shareholders in Targovax Oy in Finland and Targovax ASA in Norway.

## AUTHOR CONTRIBUTIONS

LK and A‐SWM conceptualized the study. LK and A‐SWM gave the methodolgy. LK provided software, worked on validation, and conducted formal analysis. Data LK and A‐SWM did data curation. LK and A‐SWM wrote the orginal draft and reviewed and edited it. LK visualized. LK and AS supervised the study. LK and A‐SWM were responsible for project administration. LK acquired funding.

## Supporting information

Supporting information
