# Supplementary material for: Chimeric oncolytic Ad5/3 virus replicates and lyses ovarian cancer cells through desmoglein‐2 cell entry receptor
Source: J Med Virol. 2020 Feb 3;92(8):1309–15. doi: 10.1002/jmv.25677 (PMC7496614; doi:10.1002/jmv.25677)
Supplement: Supplementary file 1 — Supporting information [file JMV-92-1309-s001.docx]

**Supplementary Table S1**. Cell lines, media, and reagents.

| **Cell lines** | **Doubling time** | **Media (all had 1% Penicillin / Streptomycin)** | **% Fetal Bovine Serum (FBS)** |
| --- | --- | --- | --- |
| **A2780** | 1 day | RPMI1640 | 10% |
| **OAW42** | 2 days | DMEM, 20 IU insulin, | 10% |
| **OVCAR3** | 3 days | RPMI1640 | 20% |
| **OV-90** | 7 days | MCDB: Medium 199 in 50:50 | 15% |
| **Products** | **Supplier** | **Item number** | - |
| DMEM | Sigma-Aldrich | R8758 | - |
| Insulin bovine | Sigma-Aldrich | I0516-5ML | - |
| MCDB 105 | Sigma-Aldrich | 117-500 | - |
| Medium 199 | Sigma-Aldrich | M4530-6X500ML | - |
| Penicillin Streptomycin | Sigma-Aldrich | P4333 | - |
| RPMI 1640 | Sigma-Aldrich | R8758-500ML | - |
| **Antibodies** | | | |
| Anti-CAR antibody | Thermo Fisher | PA5-12476 | - |
| Anti-CD46 recombinant human | Miltenyi Biotec | 130-104-559 | - |
| Anti-desmoglein-2 rabbit monoclonal antibody | Thermo Fisher | 12-9159-42 | - |
| Anti-rabbit alexa fluor 488 secondary antibody | Abcam | Ab150077 | - |
| **Other reagents** | | | |
| MTS assay | Abcam | ab197010 | - |
| Phosphate Buffer Saline PBS | Sigma-Aldrich | D8537 | - |
